# Bayesian inference reveals positive but subtle effects of experimental fishery closures on marine predator demographics

**DOI:** 10.1098/rspb.2017.2443

**Published:** 2018-01-17

**Authors:** Richard B. Sherley, Barbara J. Barham, Peter J. Barham, Kate J. Campbell, Robert J. M. Crawford, Jennifer Grigg, Cat Horswill, Alistair McInnes, Taryn L. Morris, Lorien Pichegru, Antje Steinfurth, Florian Weller, Henning Winker, Stephen C. Votier

**Affiliations:** 1 Environment and Sustainability Institute, University of Exeter, Penryn Campus, Penryn, Cornwall TR10 9FE, UK; 2 FitzPatrick Institute of African Ornithology, DST-NRF Centre of Excellence, University of Cape Town, Rondebosch, Cape Town 7701, South Africa; 3 School of Biological Sciences, University of Bristol, Life Sciences Building, 24 Tyndall Avenue, Bristol BS8 1TQ, UK; 4 H. H. Wills Physics Laboratory, University of Bristol, Tyndall Avenue, Bristol BS8 1TL, UK; 5 Animal Demography Unit, Department of Biological Sciences, University of Cape Town, Private Bag X3, Rondebosch 7701, South Africa; 6 Marine Research Institute, Department of Biological Sciences, University of Cape Town, Private Bag X3, Rondebosch 7701, South Africa; 7 Department of Environmental Affairs (DEA), PO Box 52126, Cape Town 8000, South Africa; 8 Institute of Biodiversity, Animal Health and Comparative Medicine, University of Glasgow, Glasgow G12 8QQ, UK; 9 DST/NRF Centre of Excellence at the FitzPatrick Institute of African Ornithology, Institute for Coastal and Marine Research and Department of Zoology, Nelson Mandela University, Port Elizabeth, South Africa; 10 Seabird Conservation Programme, BirdLife South Africa, PO Box 7119, Roggebaai, 8012 Cape Town, South Africa; 11 RSPB Centre for Conservation Science, Royal Society for the Protection of Birds, David Attenborough Building, Pembroke Street, Cambridge, Cambridgeshire CB2 3QZ, UK; 12 Department of Agriculture, Forestry and Fisheries (DAFF), Private Bag X2, Roggebaai, 8012 Cape Town, South Africa

**Keywords:** African penguin, Benguela ecosystem, fishing closures, forage fish, marine protected areas, seabird–fisheries interactions

## Abstract

Global forage-fish landings are increasing, with potentially grave consequences for marine ecosystems. Predators of forage fish may be influenced by this harvest, but the nature of these effects is contentious. Experimental fishery manipulations offer the best solution to quantify population-level impacts, but are rare. We used Bayesian inference to examine changes in chick survival, body condition and population growth rate of endangered African penguins *Spheniscus demersus* in response to 8 years of alternating time–area closures around two pairs of colonies. Our results demonstrate that fishing closures improved chick survival and condition, after controlling for changing prey availability. However, this effect was inconsistent across sites and years, highlighting the difficultly of assessing management interventions in marine ecosystems. Nevertheless, modelled increases in population growth rates exceeded 1% at one colony; i.e. the threshold considered biologically meaningful by fisheries management in South Africa. Fishing closures evidently can improve the population trend of a forage-fish-dependent predator—we therefore recommend they continue in South Africa and support their application elsewhere. However, detecting demographic gains for mobile marine predators from small no-take zones requires experimental time frames and scales that will often exceed those desired by decision makers.

## 1. Introduction

Quantifying the ecological consequences of fishing is one of the greatest challenges in marine conservation because of the pervasive threat fisheries pose to biodiversity [1]. About one-third of all landings are forage fish (small, schooling pelagic fish) [2], yet they are amongst the least well understood stocks. The short lifespan and planktivorous diet of forage fish causes their biomass to fluctuate more than other commercially exploited species [3]. This has also led to the orthodoxy that, relative to environmental variability, fishing mortality is generally insufficient to have meaningful impacts on dependent predators [4,5]. By contrast, some studies reveal that these fisheries *are* capable of lowering prey abundance or density to levels that affect the foraging and breeding behaviour of predators [6–10]. However, evidence for population-level impacts is rare [9], and inference is clouded by complex interactions between predators, their prey and fisheries [8,11,12].

There is a pressing need to determine definitively whether competition with forage fisheries contributes to the ongoing declines of threatened marine predators and—if so—whether or not marine protected areas (MPAs), or no-take zones (time–area closures), offer a useful mitigation option [12–15]. Management experiments using time–area closures to separate the potential effects of environmental variability and direct fishing impacts thus have global policy relevance [6,12–14]. However, they are rarely undertaken on the necessary scale and key challenges remain in assessing their impacts [9,12]. Here, we use a before–after, control–impact (BACI) experiment and Bayesian inference to address three of these challenges. Firstly, there is a need to understand the uncertainty associated with measuring predators' responses to fishery closures in light of species-specific responses to prey availability [12]. Bayesian approaches use probabilities to represent uncertainty, which is generally more intuitive than frequentist statistics [16] and more illuminating where complex ecological interactions occur [11]. Secondly, with threatened species, data deficiency can hamper the determination of objectively derived, biologically meaningful demographic responses [11]. Thirdly, such problems make it difficult to provide robust assessments in the short time frames desirable for management [12].

In Southern Africa, there is potential for competition between the sardine *Sardinops sagax* and anchovy *Engraulis encrasicolus* purse-seine fisheries and rapidly declining populations of endemic seabirds [17]. This led the South African government to initiate alternating, experimental fishing closures around two pairs of African penguin *Spheniscus demersus* breeding colonies in 2008 [8,9] (table 1). Reductions in penguin foraging effort and improvements in chick survival were noted in initial assessments of these closures [8,9]. However, these were restricted to 2 years of closure [9] and a single colony [8], and the magnitude and nature of these effects made it difficult to ascertain whether these small-scale, short-term fishing closures would generate meaningful long-term demographic benefits [9,18]. Given the importance of the underlying environmental conditions in driving penguin demography [4,18], it is unsurprising therefore that the conservation value of these closures relative to the socio-economic costs of restricting fishing—and so whether they should remain in place—is hotly debated [19–21].

**Table 1.** Schedule of purse-seine fishing closures around the four study sites. C = 20 km radius around the island was closed to purse-seine fishing, O = fishing was permitted within the 20 km radius.

Island	2008	2009	2010	2011	2012	2013	2104	2015
Dassen Island	C	C	O	O	O	O	C	C
Robben Island	O	O	O	C	C	C	O	O
St Croix Island	O	C	C	C	O	O	O	C
Bird Island	O	O	O	O	C	C	C	O

**Figure 1.**
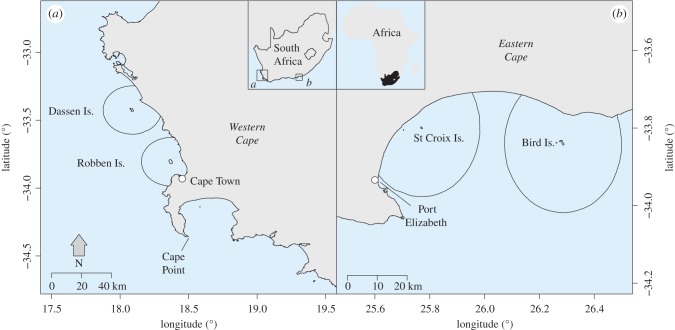
(*a*) The Western Cape of South Africa, showing Dassen Island and Robben Island in relation to Cape Town and (*b*) the Eastern Cape, showing St Croix Island and Bird Island in relation to Port Elizabeth. The 20 km radius around each island that was periodically closed to purse-seine fishing is shown as a black circle (see closure schedule in table 1). (Online version in colour.)

Here, we use data from two pairs of proximate island colonies spanning 8 years (2008–2015) before and after, with and without purse-seine fishing closures in place (a BACI design; figure 1 and table 1). We focus on two metrics of penguin breeding performance that vary with local prey availability; chick body condition and chick survival to fledging [22,23]. We also consider whether changes in these metrics can be objectively linked to population change [9,24]. This is both a requirement for their continued use as bio-indicators in fisheries management in South Africa [25], and a consideration for global best practice when assessing fisheries–seabird competition [12]. Our aims were to (i) determine whether we could detect changes in the penguin responses in the absence of fishing (closures) and, if so quantify the effect size and its associated uncertainty; (ii) assess whether effects sufficient to increase population growth rates (*λ*) by more than 1% were evident. This is the threshold considered indicative of demographic impact in a South African management context [26]; and (iii) consider whether additional years of simulated experimental closures (using data resampling and Bayes rule) would provide greater clarity for management decisions by substantially reducing the uncertainty associated with any effects. The results are discussed in the context of requirements for future experimental fishery closures and options for adaptive management in South Africa and globally.

## 2. Methods

### (a) Study sites and period

We used data from two sets of paired islands: Robben Island (33°48′ S, 18°22′ E) and Dassen Island (33°25′ S, 18°06′ E) in South Africa's Western Cape province and St Croix Island (33°47′ S 25°46′ E) and Bird Island (33°50′ S, 26°17′ E) in the Eastern Cape province (figure 1). Between 2008 and 2015, a purse-seine fishing closure was alternated between each island in the pair (table 1). The closures comprised a 20 km radius around each penguin colony (figure 1), designed to encompass the foraging range of chick-rearing penguins [8,22]. During the study period, the penguin populations in the Western Cape declined from approximately 5700 breeding pairs in 2008 to approximately 2100 in 2015 at Dassen Island, and from approximately 4200 to approximately 1200 pairs at Robben Island ([17]; DEA, unpublished data). In the Eastern Cape, the penguin populations remained stable over the same period: approximately 7700 pairs at St Croix Island and approximately 2800 at Bird Island ([17]; DEA, unpublished data).

### (b) Penguin response data

We measured chick condition at all four islands between 2008 and 2015. Nests were selected at random and chicks were measured for head length (tip of the bill to back of the skull; ±0.1 mm) using Vernier calipers, and mass (±10 g) using electronic or spring balances. Measurements were made approximately 5–10 days apart from January to December on Dassen Island (which has an extended breeding season), and between March and November at the other sites. We estimated body condition using a species-specific index based on a cohort of chicks with head lengths greater than 75 mm that survived to fledging [27]; smaller chicks (generally lesser than or equal to 20 days old) were excluded from our analysis.

Data on chick survival were collected at the Western Cape islands from 2008 to 2015. Marked nests were checked at target intervals of 4–7 days at Robben Island throughout the main breeding season (March to October), and 5 days at Dassen Island throughout the whole year. We recorded the presence and number of chicks at each visit, calculated the number of days exposed to potential mortality (nestling days) and recorded whether mortality occurred (=1) or not (=0) [9,23]. Where monitoring was curtailed before the nesting attempt had been completed, we considered the data to be right censored at the last time a chick was seen [23] (see the electronic supplementary material).

### (c) Fish biomass data

We used hydro-acoustic survey estimates of sardine and anchovy biomass in South Africa from 2008 to 2015 [28,29] as a predictor to control for any temporal trends or changes in prey availability [9]. Annual surveys in May estimate the biomass of recruit (age 0) fish, while surveys during November estimate the adult sardine and anchovy biomass, excluding age 0 juveniles (see electronic supplementary material). For the Western Cape islands, we used the adult sardine biomass west of Cape Agulhas estimated from the November survey of the previous year and the anchovy recruit biomass (which predominately occurs west of Cape Agulhas [30]) in the year in which chick condition and survival to fledging were measured [9,23]. Based on their location outside of the usual range of anchovy recruits [30], for the Eastern Cape islands we used both the adult sardine and anchovy biomass east of Cape Agulhas from the November survey of the previous year. No catches were reported within the closed areas, though fishing continued outside [8,9]. We did not use data on catches taken beyond the closed areas to account for fishing pressure near colonies here because, as noted elsewhere, correlations between catch and biomass data can bias model parameter estimates [11,31].

### (d) Estimates of closures effect size and uncertainty

For chick condition, we implemented a linear-mixed model structure, with random intercepts for the month in which each chick was measured, nested within the monitoring year. Because access to prey resources differs [17], we modelled the Western and Eastern Cape data separately. Fixed effects were the island (Robben and Dassen, or St Croix and Bird), closure status (‘Open’ or ‘Closed’ to fishing, table 1), an interaction between island and closure status, as well as additive effects of sardine (S) and anchovy (A) biomass (to account for changing prey availability driven by factors other than fisheries effects). The full model took the form2.1
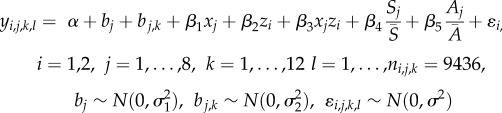
where 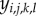 is the chick condition for each individual chick (*l*), in month *k* of year *j* at island *i*; *α* is the intercept; *b_j_* denotes the year random effect and 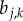 the month random effect (nested in *b_j_*); the *β*'s are the coefficients to be estimated for the fixed effects; *x_j_* is a binary covariate for the island closure effect (‘Open’ = 0, ‘Closed’ = 1); *z_i_* is a binary covariate denoting to which island a chick belongs (e.g. Dassen = 0, Robben = 1); *S_j_* and *A_j_* are sardine and anchovy (respectively) biomass estimates associated to year *j* (see above for details) and 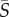 and 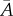 the mean biomass of each species over the years considered; 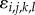 is the residual error; and the variance terms (*σ*) for the random effects and residual error were estimated from the data.

For chick survival, we estimated failure rates (deaths/unit time of exposure or hazard functions) for ‘Open’ or ‘Closed’ years using an exponential error distribution and used an exponential distribution to transform these failure rates into chick survival estimates [9,23]. We used nest identity within year to specify a hierarchical shared frailty term (analogous to a random effect [32]); i.e. the survival rates of chicks within the same nest are considered non-independent. The hazard function (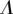) was estimated as2.2
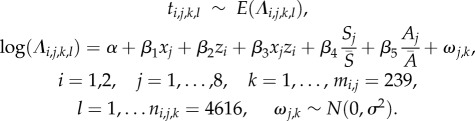



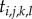
 denotes the observed time of exposure for each chick (*l*), in nest *k*, in year *j*, at island *i*; 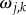 denotes the shared frailty term and all other parameters are as in equation (2.1).

All models were implemented using Markov chain Monte Carlo (MCMC) estimation in JAGS (v. 4.1.0) via the ‘jagsUI’ library (v. 1.3.7) for program R v. 3.2.1. The uninformative priors were 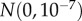 for estimated means (where 10^−7^ is precision) and 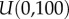 for standard errors (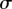), with the precision specified as 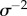 [33]. For chick condition, we ran three chains of 55 000 samples, discarded the first 5000 as burn-in and drew inference from the rest of the chains with no thinning. To account for the additional complexity of the chick survival model, three chains of 3 million samples were run, discarding the first 1 million as burn-in and thinning to every 10th observation to increase the effective MCMC sample size for the same amount of computer memory. For both chick condition (equation (2.1)) and survival (equation (2.2)), the explanatory variables included in the full model (electronic supplementary material, table S1) were those considered relevant based on our prior knowledge of the system [9,23] (but see the issue of catch-biomass correlation noted above). However, because our focus was on estimating the effect of fisheries closures, when the 95% credible intervals (CI) for the island/closure interaction estimate overlapped zero, we considered there to be no evidence for island-specific effects and, based on parsimony, dropped this parameter from the final model used for inference (electronic supplementary material, table S1). All models were checked for convergence visually and using Gelman–Rubin diagnostics (all 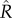 values ≤1.01).

### (e) Simulation of data for additional experimental years

To examine how the uncertainty associated with closure effects might respond to additional years of experimental closures, we simulated an extended time series of chick condition data for the Western Cape (the largest dataset). Data were imputed based on a future sequence of 3 years ‘Closed’, followed by 3 years ‘Open’ at each island (table 1). To produce each dataset, the Western Cape chick condition model (equation (2.1)) was rerun with thinning to every 50th observation to subsample 1000 iterations of each MCMC chain and generate one posterior distribution for each of the *l* = 9436 observed chick measurements. Next, we simulated a sample size (number of chicks measured) for each future year and island (*n_sy_*) using a random draw from a uniform distribution bounded by the observed sample sizes at each island (Dassen Island: *U*(255,947); Robben Island: *U*(323,1176)). For each year, and each island, we randomly drew (with replacement) *n_sy_* chick condition values from the posterior distributions in a stratified manner according to whether that island was scheduled to be ‘Open’ or ‘Closed’ that year. Each sample was therefore a random draw from a posterior distribution (corresponding to each observation *l*) with a mean equal to the original observation and a variance specified by the data. Each simulated estimate was then assigned to the calendar month of the corresponding observation [16]. New data were simulated for 4, 7 and 10 years (electronic supplementary material, figures S2 and S3) and attached to the observed data. The model in equation (2.1) was fitted to these new datasets, excluding the biomass predictors (uninformative in the original analysis; electronic supplementary material, table S1 and figure S1). To examine how these new data influenced the probability of detecting effects, we compared the posterior means and 95% CI of the *β* terms (see equation (2.1)) with those from the original model fit. Finally, to confirm that any changes were not an artefact of the sample used in each case, we repeated the resampling process to generate 1000 new datasets for each of 4, 7 and 10 additional years. We then compared the parameter estimates from the respective JAGS model to the mean effect size and 95% quantiles from fitting 1000 frequentist models (using ‘nlme’ v. 3.1-122) to each new dataset (see electronic supplementary material).

### (f) Population model projections

We used a Bayesian projection model with a demographic structure and parameter values based on previous African penguin models [9,34] (electronic supplementary material, table S2). Adult survival (*ϕ*_a_ = 0.743) was deterministic to allow for clear comparisons between different scenarios for juvenile (*ϕ*_j_) and chick survival (*ϕ*_c_). We modelled *ϕ*_j_ and *ϕ*_c_ as stochastic using observed means and standard deviations (s.d.) (electronic supplementary material, table S2). The baseline run was parameterized to represent ‘Open’ to fishing at both islands; *ϕ*_c_ was set at the mean (±s.d.) value estimated for all ‘Open’ years at both islands, and *ϕ*_j_ = 0.194 (±0.117) based on published estimates [35]. We modelled means ±95% Bayesian CI using three MCMC chains (225 000 samples, burn-in of 25 000, no thinning), confirmed unambiguous model convergence (all 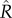 < 1.01), and compared the population projections (±95% CI) to census data from Robben and Dassen islands between 2004 and 2015 (electronic supplementary material, figure S7).

To assess the effect of fishery closure, we modified the priors assigned to *ϕ*_c_ and *ϕ*_j_ according to the measured ‘closure effect’ on chick condition and survival and examined whether the observed effects would improve population growth rates by more than 1% above the baseline rate (Δ*λ* ≥ 1%). For Robben Island, we also assessed the impact of the simulated 10 additional experiment years of chick condition data on the uncertainty associated with Δ*λ*. For chick survival (*ϕ*_c_), we used priors with an island-specific mean and s.d. estimated for all closed years (from equation (2.2)). In the absence of species-specific data to link improvements in chick body condition directly to juvenile (*ϕ*_j_) or chick survival (*ϕ*_c_), we used observed relationships between mass at fledging and first-year survival in macaroni penguins *Eudyptes chrysolophus* [24], and between mass at fledging and chick body condition in African penguins [34] (electronic supplementary material, figure S8). We assessed the validity of this approach against an assumption of proportional change in *ϕ*_j_ with changes in body condition. These modified models were run as the baseline model, but we used the individual Robben Island (1216 breeding pairs) and Dassen Island (2140 breeding pairs) populations in 2015 as starting populations to model the size of the breeding populations in 2025 and 2035 (±95% CI) to compare with the baseline model. See electronic supplementary material for model details.

## 3. Results

### (a) Estimates of closure effect size and uncertainty from observed and simulated data

#### (i) Western Cape

**Figure 2.**
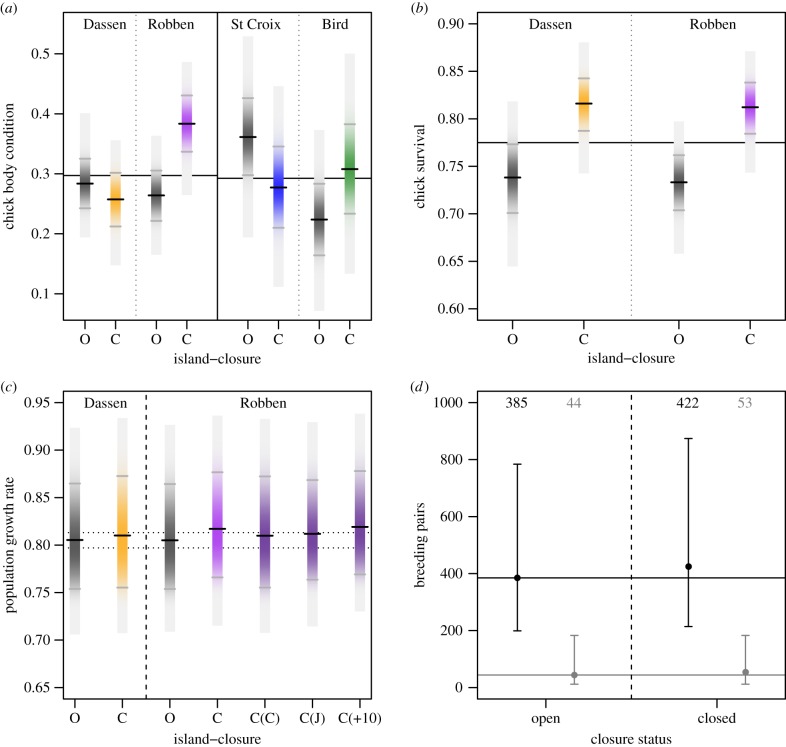
Posterior distributions (in *a–c* only), means and 95% credible intervals (CI) at Dassen Island, Robben Island, St Croix Island (*a* only) and Bird Island (*a* only) for years where fishing was permitted (‘Open’ or ‘O’) or not permitted (‘Closed’ or ‘C’) for (*a*) chick body condition, (*b*) chick survival, (*c*) population growth rates (*λ*) and (*d*) predicted population sizes (at Dassen and Robben islands combined). In *a*–*c*, ‘Open’ results are shown in black, ‘Closed’ are in orange for Dassen, purple for Robben, blue for St Croix, green for Bird. Black tick marks denote the posterior mean (calculated at mean anchovy and sardine biomass), grey ticks the 95% CI and grey polygons the range of the posterior distribution. The solid black lines show overall mean chick condition (*a*) and chick survival (*b*) rates for all chicks across all years (2008–2015) at each island pair. In *c*, dashed black lines show a 1% change in baseline *λ*, ‘C(C)’ indicates a model run for Robben Island where only chick survival (*ϕ*_c_) was improved, ‘C(J)’ where only juvenile survival (*ϕ*_j_) was improved and ‘C(+10)’ where the chick condition effect came from the model using 10 years of additional simulated data (figure 3). In *d*, mean (points) and 95% CI (error bars) of predicted population size in 2025 (black) and 2035 (grey) are based on *λ*-values in (*c*) and a starting population at the stable-age distribution; each posterior mean is given at the top of the plot. (Online version in colour.)

**Figure 3.**
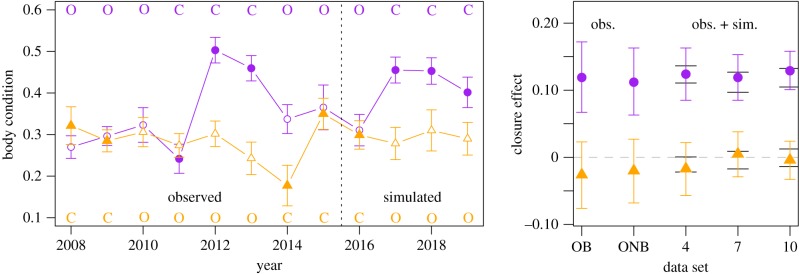
Left panel: observed (2008–2015) and simulated (2016–2019) annual means and approximate 95% confidence intervals for chick condition at Dassen Island (orange triangles) and Robben Island (purple circles). The ‘closure’ status for each year is indicated by open or closed symbols and in island-specific colours; ‘O’ = purse-seine fishing was permitted around that island in that year, ‘C’ = purse-seine fishing was excluded within a 20 km radius. Right panel: The posterior means and Bayesian 95% credible intervals for the estimated effect of closure to fishing on penguin chick condition at Dassen Island and Robben Island. An effect size above zero (dashed grey line) means higher chick condition on average when fishing was restricted with 20 km of that island, a negative effect size the opposite. From the left, the effect sizes are for the model fit to the observed data (2008–2015) including sardine and anchovy biomass estimates (accounting for prevailing environmental conditions; OB); the model refit to the observed data without sardine and anchovy biomass (ONB); the model refit to the observed data plus a case including 4 years of simulated data (4, see left panel); a case including 7 years of simulated data (7) and a case including 10 years of simulated data (10). Long black ticks on the cases including simulated data show the 95% quartiles from frequentist model fits to 1000 additional simulations. (Online version in colour.)

*Chick condition*: Based on the observed data and the full model (electronic supplementary material, table S1), mean condition at Dassen Island was 0.284 (95% CI: 0.242–0.325) during ‘Open’ years and 0.257 (0.212–0.302) during ‘Closed’ years at mean fish biomass, or 9% lower without fishing (figure 2). However, the 95% CI for this effect included zero, with 15% of iterations actually yielding a positive closure effect (figure 3). Adding more years of simulated data reaffirmed this null effect, rather than confirming a weak negative effect as the uncertainty was reduced; the mean effect size shifted closer to zero, from −9% in the observed data to −2% with 10 years of simulated data (figure 3; electronic supplementary material, figure S5).

By contrast, at Robben Island chick condition significantly and unambiguously improved by 45% without fishing, from 0.264 (0.222–0.305) during ‘Open’ years to 0.383 (0.336–0.430) during ‘Closed’ years (figure 2) based on the observed data. The mean effect of closure on chick condition remained essentially unchanged as more years of simulated data were added, varying between+41% and +45% (figure 3; electronic supplementary material, figure S3 and S5).

In all cases, the simulated datasets produced appropriate means and distributions relative to the observed data (figure 3; electronic supplementary material, figure S2–S4), and the mean effect sizes estimated in JAGS lay within the 95% quantiles from the 1000 ‘nlme’ model fits, demonstrating that the simulated datasets were robust to sampling variation and not just artefacts of the particular run used (figure 3; electronic supplementary material, figure S6).

*Chick survival:* For chick survival, there was no support for the island/closure interaction term (electronic supplementary material, table S1). The simplified model estimated that fisheries closures improved chick survival at Dassen Island by 11.2%, from 0.738 (0.708–0.773) during ‘Open’ years to 0.816 (0.787–0.843) during ‘Closed’ years (at mean prey biomass). Fishery closures also increased chick survival at Robben Island by 10.8% from 0.733 (0.704–0.762) in ‘Open’ years to 0.812 (0.784–0.838) in ‘Closed’ years (figure 2). There was essentially no uncertainty in the differences between the means for either island (non-overlapping 95% CI, figure 2) and the posterior of the fishery closure main effect did not overlap zero (electronic supplementary material, table S1).

#### (ii) Eastern Cape

*Chick condition*: Mean condition at St Croix Island was 0.361 (0.298–0.4260) in ‘Open’ years and 0.277 (0.210–0.345) in ‘Closed’ years. At Bird Island, mean chick condition was 0.224 (0.164–0.283) and 0.308 (0.234–0.383) in ‘Open’ and ‘Closed’ years, respectively. Fishery closures appeared to generate weak, opposing effects at these islands; i.e. condition increased (0.084, 95% CI: 0.004–0.164) at Bird Island and decreased (−0.084, 95% CI: −0.162 to −0.007) at St Croix Island. However, the 95% CI for both sets of scenarios overlapped, suggesting that overall, the closure had no impact in this case (figure 2) and therefore further simulations were redundant.

### (b) Population model projections

The baseline population growth rate (*λ*) at Dassen and Robben Island, estimated with open fisheries, demographic stochasticity and parameter uncertainty on *ϕ*_c_ and *ϕ*_j_, was *λ* = 0.805 (95% CI: 0.754–0.864). This was comparable to the equivalent deterministic Leslie matrix estimate, *λ* = 0.809 and the projections reproduced the observed population trajectory (electronic supplementary material, figure S7). When *ϕ*_c_ and *ϕ*_j_ were increased by the observed effect sizes of fishing closure, *λ* improved at both Dassen Island (*λ* = 0.810, 95% CI: 0.755–0.873) and Robben Island (0.817, 0.766–0.877); in the latter case Δ*λ* > 1% compared to the baseline model (figure 2). However, for model runs at Robben Island where either *ϕ*_c_ or *ϕ*_j_ were increased separately by the observed effect sizes, Δ*λ* did not exceed this 1% threshold. Furthermore, adding 10 years of additional simulated experimental data did little to reduce the uncertainty in the demographic impact (figure 2). The projected population in 2025 was approximately 10% larger than the baseline when both closure effects (on *ϕ*_c_ and *ϕ*_j_) at Robben Island and the chick survival effect at Dassen Island were modelled, and approximately 20% higher by 2035 (figure 2). The modelled population, however, continued to decline under all scenarios, but was only two-thirds as likely to drop below 500 pairs by 2025 with the modelled closure effect (16%) than without it (24%; figure 2).

## 4. Discussion

Until now it was unclear whether forage fisheries deplete prey sufficiently to have population-level effects on marine predators [5,12]. Our results reveal that fisheries closures improved chick condition and survival at one African penguin colony sufficiently to improve their population trajectory. Accordingly, we recommend that these closures be retained. However, even with a BACI design, an 8-year time frame, and complex analytical approaches, the effects were subtle and inconsistent, highlighting the extremely challenging nature of quantifying forage fishery impacts. Although we studied metrics that vary with local prey availability [22,23], we only detected fisheries effects with certainty in three of six cases, and at two of four islands. Those designing similar closures in future should consider this when setting (and stating) their expectations for sites and metrics to study [12], and that the required experimental periods (perhaps decades) may conflict with a desire for rapid management action.

Our results also underline the difficulty of controlling for changes in the underlying environmental conditions in a dynamic ecosystem, even when measures of prey availability are available. In a scenario where fishing had no effect and the prey availability estimates included in the models were unable to perfectly account for changes due to a common environmental driver, we would have expected opposing positive and negative signs in the mean differences between ‘Open’ and ‘Closed’ years at the two islands in a pair. This is exactly the case for the effects on chick condition, highlighted by the matching absolute effect size at Bird and St Croix islands (figure 2). It may be that our measures of prey abundance did not fully account for the local variation in prey availability around the island pairs [23]. We also did not control for the presence of fishing in close proximity to the closed areas (fishing the line), which can influence MPA efficacy for mobile fish and their predators [8,36]. Both issues increase the difficulty of detecting a closure signal from the ecological ‘noise’ and could explain the apparent absence of effects on chick condition at Dassen Island or in the Eastern Cape. In addition, we cannot confirm biomass removal, rather than disturbance (of shoaling or foraging behaviour), as the mechanism of competition without concurrent behavioural data on fish and penguins [12]. This is difficult to collect at the relevant scales [22] (but see [37]). However, if prey availability is not accounted for adequately, a few extreme years or temporal trends could easily confound environmental variability and fishing impacts when experimental periods are short [8,9], even with a BACI design. Future experimental closures, both in South Africa and elsewhere, would benefit from fisheries-independent assessments of prey availability on a scale relevant to the focal predator [38]. The above notwithstanding, the magnitude of improvement in chick condition at Robben Island, and the consistently higher chick survival during closed years at both Western Cape islands provides strong evidence for a fisheries effect over and above that of a common environmental driver.

Although our aim was to quantify the uncertainty associated with detecting penguin responses to the closures, none of the posterior distributions for the closure effect on chick survival at the two Western Cape colonies fell below zero (i.e. there really was no uncertainty in this instance). For chick body condition at Robben Island, less than 1% of the posterior distribution was negative and this small uncertainty disappeared with an additional 3 years of simulated data (electronic supplementary material, figure S5). At the other islands, the effects were essentially indistinguishable from zero, or became so with 10 years of simulated data at Dassen Island. The mean effect sizes were also relatively robust to the addition of simulated data (figure 3), so inference is unlikely to be altered in the short to medium term. Crucially though, the observed closure effects at Robben Island increased the modelled population growth rate sufficiently to exceed the criteria for a meaningful demographic effect set by fisheries management in South Africa (Δ*λ* > 1%). However, the uncertainty around this demographic effect was high and decreased little with 10 additional years of simulated sampling (figure 2). Moreover, the observed impacts on chick survival alone were insufficient to exceed the 1% threshold (it also required a change in juvenile survival). This highlights the importance of considering whether combined, or compound, effects of fishing are likely to operate on demographic rates on a case by case basis [5].

Overcoming uncertainty in the potential demographic impact of fisheries closures is likely to remain difficult, even in systems where seabird–fisheries interactions are relatively well understood [12]. It is often difficult or time-consuming to acquire reliable data on important demographic processes, such as immature or adult survival [35], and more readily accessible behavioural data—for example, on foraging effort—is challenging to link to demography [10,12]. Accordingly, without detailed knowledge of the underlying ecosystem, clear cut, consistent demographic responses across focal sites and species are unlikely to arise from experimental fisheries closures in desirable time frames for management (years not decades; [6,11,12]). Although continuing the closures will affect the South African purse-seine industry, estimates vary widely from less than 1% to approximately 9% of total annual catches for closures at both Western Cape colonies [39]. Any costs also need to be weighed against the high socio-economic value of penguin-based ecotourism [40] (our study colonies hold approximately 60% of South Africa's breeding penguins) and the likelihood that spatial protection around these islands would benefit wider marine biodiversity, including other threatened marine predators [38]. Conservation actions are sometimes deferred because of doubt or fear of failure, but delay can increase the risk of extirpation or extinction [41,42]. In short, although uncertainty is likely to remain, it can be quantified, understood and formally incorporated into management decision making [42]. In light of this, we strongly recommend a precautionary approach when impacts on components of the demographic process can be measured; management should then proceed in an adaptive framework [13,42], with spatial protection the default, particularly for populations in severe decline.

Finally, our results highlight the need to carefully consider the value of small-scale protected areas for long-lived, motile marine species where benefits to adult survival may be subtle [9,34]. In our projections, the population continued to decline markedly under all scenarios. If low first-year and adult survival persists, which may depend more on wide-scale rather than local processes [34,35], the benefits of small-scale protected areas may be limited [18]. This will not be the case in all situations and while broad-scale conservation actions (e.g. catch quotas, bycatch reduction) will be needed in concert [10,34], they are often more difficult, time-consuming or costly to implement than spatial protection. Without prompt action, the penguin population off South Africa's west coast could be functionally extinct by 2035 (less than 50 pairs; figure 2). Despite the ecological ‘noise’, our models indicate that small-scale fishing closures will improve that outlook; combining this approach with broad-scale, ecosystem-based fisheries management would ensure an even brighter future for African penguins and many other threatened marine predators.

## Supplementary Material

Detailed methods, additional results and model code.

## Data accessibility

The data are in the electronic supplementary material or Dryad digital repository: http://dx.doi.org/10.5061/dryad.d4977 [43].
