# Correction to ‘Bayesian inference reveals positive but subtle effects of experimental fishery closures on marine predator demographics'

**DOI:** 10.1098/rspb.2021.2129

**Published:** 2021-11-24

**Authors:** Richard B. Sherley, Barbara J. Barham, Peter J. Barham, Kate J. Campbell, Robert J. M. Crawford, Jennifer Grigg, Cat Horswill, Alistair McInnes, Taryn L. Morris, Lorien Pichegru, Antje Steinfurth, Florian Weller, Henning Winker, Stephen C. Votier

*Proc. R. Soc. B* **285**, 20172443. (Published online 17 January 2018) (doi:10.1098/rspb.2017.2443)

It has recently come to our attention that we made a coding error while implementing the hierarchical mixed-models describing chick condition and chick survival (equations (2.1) and (2.2), respectively) in ‘Bayesian inference reveals positive but subtle effects of experimental fishery closures on marine predator demographics' [1]. This error meant that the nested random effect described in equation (2.1) and the hierarchical shared frailty term described in equation (2.2) were not implemented correctly in the original analysis. This resulted in our reporting the model parameter estimates with higher precision than should have been the case (table 1 and figure 1). Here we present corrected results (table 1 and figure 1) based on correctly specified models in JAGS [2] applied to the datasets used in our original analysis. Our error, which pertained to the specification of the structure of the priors for the nested random effects (see electronic supplementary material), influenced the derived parameter estimates of the mean chick condition or chick survival during closed and open years (figure 1) more (standard deviations are between 29% and 285% larger in the corrected results) than the estimates for the regression coefficients representing the island effect (–0.8 to 9%), closure effect (4–8%) or their interaction (6–8%). These changes do not alter the predicted influence of the fisheries closures on the population dynamics of endangered African penguins *Spheniscus demersus*.

**Table 1.** Responses of African penguins to purse-seine fishing closures around four study sites based on the incorrectly specified models in our original analysis [1] (original results) and based on models with the corrected random-effects structures (corrected results). Unless otherwise specified, the posterior mean and 95% credible intervals are shown. Notes: DI, Dassen Island; RI, Robben Island; BI, Bird Island; SI, St Croix Island; *, the closure effect size is in log-space where the intercept (log(daily mortality rate)) = –5.503, thus the closure effect represents a change in the daily mortality rate from 0.0041 in open years to 0.0027 in closed years; *ϕ_c_* = chick survival; *ϕ_j_* = juvenile survival (modified by the closure effect on chick condition); *λ* = the population growth rate. Closed = 20 km radius around the island was closed to purse-seine fishing, O = fishing was permitted within the 20 km radius. Pop. = population size (in numbers of breeding pairs).

Western cape chick condition									
	closure effect	DI open	DI closed	% change	interaction effect	mean difference at RI	RI open	RI closed	% change
original results	−0.026 (−0.076 to 0.023)	0.284 (0.242–0.325)	0.257 (0.212–0.302)	−9% (−25 to 9%)	0.146 (0.052–0.241)	0.120 (0.068–0.172)	0.264 (0.222–0.305)	0.383 (0.336–0.430)	45% (23–73%)
corrected results	−0.032 (−0.086–0.020)	0.287 (0.232–0.346)	0.255 (0.194–0.312)	−11% (−28–8%)	0.157 (0.057–0.260)	0.125 (0.070–0.180)	0.262 (0.203–0.317)	0.387 (0.327–0.450)	48% (24–81%)

Eastern cape chick condition									
	closure effect	BI open	BI closed	% change	interaction effect	mean difference at SI	SI open	SI closed	% change
original results	0.084 (0.004–0.164)	0.224 (0.164–0.283)	0.308 (0.234–0.383)	38% (2–89%)	−0.168 (−0.309 to −0.027)	−0.084 (−0.162 to −0.007)	0.361 (0.298–0.426)	0.277 (0.210–0.345)	−23% (−42 to −2%)
corrected results	0.091 (0.006–0.177)	0.223 (0.133–0.313)	0.314 (0.215–0.420)	41% (2–108%)	−0.179 (−0.331 to −0.024)	−0.087 (−0.170 to −0.004)	0.365 (0.274–0.461)	0.277 (0.183–0.373)	−24% (−44 to −1%)

Western cape chick survival									
	closure effect*	di open	DI closed	% change	interaction effect	mean difference at RI	RI open	RI closed	% change
original results	−0.402 (−0.548 to −0.256)	0.738 (0.701–0.773)	0.816 (0.787–0.843)	11% (6–15%)	n.a.	n.a.	0.733 (0.704–0.762)	0.812 (0.784–0.838)	11% (7–15%)
corrected results	−0.383 (−0.536 to −0.231)	0.740 (0.615–0.836)	0.814 (0.717–0.886)	10% (5–19%)	n.a.	n.a.	0.731 (0.604–0.829)	0.807 (0.707–0.881)	10% (5–19%)

population projection models									
	baseline model (λ)	DI *ϕ*_c_ increase (λ)	RI *ϕ*_c_ and *ϕ*_j_ increase (λ)	RI *ϕ*_c_ increase (λ)	RI *ϕ*_j_ increase (λ)	Pop. 2025 open	Pop. 2025 closed	Pop. 2035 open	Pop. 2035 closed
original results	0.805 (0.754–0.864)	0.810 (0.755–0.873)	0.817 (0.766–0.877)	0.810 (0.755–0.872)	0.812 (0.764–0.868)	385	422	44	53
corrected results	0.805 (0.754–0.865)	0.810 (0.755–0.873)	0.817 (0.766–0.877)	0.810 (0.755–0.872)	0.812 (0.764–0.869)	385	421	44	53

**Figure 1.**
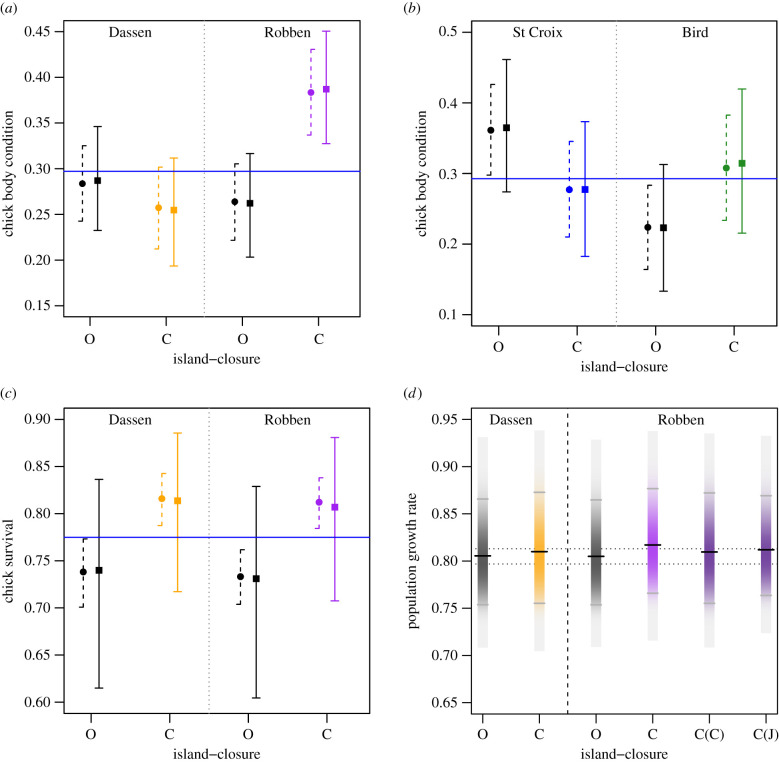
African penguin *Spheniscus demersus* chick body condition (*a* and *b*), chick survival (*c*) and population growth rate (*λ*) (*d*) at four penguin breeding colonies when a 20 km radius around the respective island was open to purse-seine fishing (O, open) or was closed to purse-seine fishing (C, closed). ‘Open’ results are shown in black, ‘closed’ are in orange for Dassen Island, purple for Robben Island, blue for St Croix Island, and green for Bird Island. In (*a*–*c*) circles and dashed lines show the published mean (calculated at mean prey biomass) and 95% credible intervals (CRI) presented in the original published paper [1], which were based on models where the hierarchical random effect (frailty term in (*c*)) was incorrectly implemented in JAGS [2], while squares and solid lines show the mean and 95% CRI based on the models with the correctly specified hierarchical random effect (month nested in year for condition, nest identity nested in year for survival) implemented in JAGS. In (*d*) black horizontal lines denote the posterior mean, grey horizontal lines the 95% CRI and the light grey extremes of each bar show the range of the posterior distribution. The dashed black lines show a 1% change in baseline population growth rate, ‘C(C)’ indicates a model run for Robben Island where only chick survival (*ϕ_c_*) was improved, ‘C(J)’ where only juvenile survival (*ϕ_j_*) was improved. These population projection models are based on the updated results (squares and solid lines) in (*a*) and (*c*): they show essentially unchanged results from the models published in the original paper [1] (table 1). (Online version in colour.)

In our original analysis, we used vague uniform prior distributions, Uniform(0, 100), to assign the standard deviations for the variance components of the random effects and residual error. In the updated analysis this choice led to poor mixing of the Markov chain Monte Carlo (MCMC) chains for the regression parameters in all models. The choice of vague priors for the variance terms associated with the hierarchical random effects can affect model convergence and inference [3,4]. However, Gelman [3] recommends using a half-Cauchy distribution with scale = 25 as a vague prior for the variance terms associated with the hierarchical random effects (and any reasonable vague prior distribution for the residual standard deviation). The probability density function for the half-Cauchy distribution with scale = 25 has its maximum value at *x* = 0, but remains high over values *x* < 50, falling off gradually beyond this point (electronic supplementary material, figure S1). Here, half-Cauchy(25) for the variance terms associated with the hierarchical random effects and Uniform(0, 10) for the residual standard deviation led to good convergence for our datasets. For the chick condition models we ran three MCMC chains of 100 000 samples, discarded the first 50 000 as burn-in and drew inference from the rest of the chains with no thinning. To account for the additional complexity of the chick survival model, we ran three chains of 200 000 samples, discarding the first 100 000 as burn-in, and thinned the chain to every second iteration. All models unambiguously converged (all $\hat{R}$ values ≤ 1.01, smallest effective sample size in any model = 721). All other methods were as described in our original analysis [1]. Unless otherwise specified, all results are posterior means ± 95% credible intervals (CRI).

The corrected results (table 1 and figure 1) yield very similar point estimates to the results published in the original paper, but with wider 95% CRI (figure 1*a–c*). In the original paper, we reported that chick condition at Robben Island improved significantly and unambiguously by 45% in the absence of fishing, and that the fisheries closures improved chick survival by approximately 11% at both Dassen Island and Robben Island (table 1), with essentially no uncertainty in the differences between the means for either island (non-overlapping 95% CRI). Both of these closure effects remain credibly different from zero at the 95% level in the corrected results and the effect sizes remain similar (48% and 10% for condition and survival, respectively; table 1). By contrast, the 95% CRI for the derived estimates of chick survival in years that were ‘open’ to fishing and ‘closed’ to fishing now overlap at both Dassen Island and Robben Island (figure 1*c*), meaning that there is more uncertainty in this case than we originally reported. This is probably because unmodelled sources of random effect variance often have little effect on the parameter estimates for the fixed effects (e.g. the closure effect) but manifest as additional residual variance [5] (i.e. the ‘missing’ uncertainty was in the residual error rather than the random intercepts).

Incorporating the corrected results into the population projection models used in the original paper did not change (apart from some MCMC rounding, table 1) the predicted population level effects (figure 1*d* here, fig. 2*c* in the original paper). This probably reflects the similarities between the point estimates and effect sizes on which the closure effects are based (shown in figure 1*a,c*) in the corrected and original analyses (table 1), and because the majority of the uncertainty in the population projection models already came from parameter uncertainty in mean juvenile survival (from fledging to age one) due to high variation in that trait over time [6]. Accordingly, our original conclusions that fisheries closures could provide subtle improvements in the population trend of a forage-fish-dependent predator are still supported by the corrected results.

## Data accessibility

The data used in both this correction and the original analyses are available from the Dryad Digital Repository: https://datadryad.org/stash/dataset/doi:10.5061/dryad.d4977 [7]. The corrected code is provided in electronic supplementary material, S2.
