# Supplementary material for: Bayesian inference reveals positive but subtle effects of experimental fishery closures on marine predator demographics
Source: Proc Biol Sci. 2018 Jan 17;285(1871):20172443. doi: 10.1098/rspb.2017.2443 (PMC5805942; doi:10.1098/rspb.2017.2443)
Supplement: Detailed methods, additional results and model code. [file rspb20172443supp1.pdf]

Electronic Supplementary Material S1

Additional methods and results for estimating the closures effect sizes and uncertainty

Penguin response data: At Robben Island, chicks were classified into five stages of development and considered to have fledged if they reached the last stage (>50% fledging plumage) [1,2]. At Dassen Island, fieldworkers recorded if chicks were in downy or fledging plumage and the apparent outcome of each nest ('Failed' or 'Fledged'). For each chick, we calculated the number of days exposed to potential mortality (nestling days) by taking the mid-points between visits and recorded whether mortality occurred (= 1) or not (= 0) [3]. In African penguins, chicks are guarded until ~40 days old, after which they may wander from nests or join crèches [4], subsequently evading detection. However, fledging does not occur until chicks are >60 days old [4,5]. Thus, we considered chicks to have died if they disappeared after <40 nestling days and no fate was clearly recorded (even if no carcass was observed). If chicks disappeared after ≥40 days, but had not been recorded in fledging plumage, we considered the monitoring to have been truncated (data were right censored) at the last time the chick was seen [2]. Accordingly, for censored observations, the time to death was imputed by the model using the interval distribution in JAGS as a random value greater than the specified censoring limit [6]. Here, we based the censoring limit for each of these chicks on the assumption that they survived for at least one more day after they were last observed in the nest.

Fish biomass data: The South African Department of Agriculture, Forestry and Fisheries conduct annually hydro-acoustic surveys to estimate the biomass of sardine and anchovy in South African waters. These surveys span a large area surrounding the penguin breeding colonies that we studied. In May, the waters between the Orange River mouth (28°38'S, 16°27'E) and Cape St. Francis (34°11'S, 24°50'E) are surveyed to estimate the biomass of recruit (age 0) anchovy – these recruit fish predominately occur west of Cape Agulhas and are scarce as far east as St. Croix and Bird Island [7]. The diet of provisioning African penguins is dominated by recruit anchovy (typically >75% by mass), with sardine present in smaller quantities (usually <6% by mass) [2,8]. Similarly, the area between Hondeklip Bay (30°19'S, 17°16'E) and Port Alfred (33°36'S, 26°53'E) is surveyed during November to estimate adult sardine and anchovy biomass (age 0 juveniles excluded). Adult sardine (in particular) and anchovy appear to be important prey for survival of non-breeding penguins [3,9,10]. Consequently, we based our choices on which biomass estimates to use in the models based on these differences in the distribution and utilization by penguins of the different age-classes of sardine and anchovy (see main text).

Table S1. Posterior means and 95% credible intervals (CI) for the fixed effects estimated to assess the impact of purse-seine fishing closures on African penguin chick condition and survival at the four study sites (one pair in the Western Cape, one pair in the Eastern Cape; figure 1). Where a model was simplified for inference, both the full and final model structure are shown.

Dataset	Model structure	Parameter	Posterior mean (SD)	95% CI	Overlap zero?
		Western Cape (Dassen and Robben islands)			
Chick Condition	Full model (Eqn 1)	Intercept (a)	0.284 (0.021)	0.242–0.325	No
		Closure main effect (β_1)	−0.026 (0.025)	−0.076–0.023	Yes
		Island main effect (β_2)	−0.020 (0.023)	−0.065–0.025	Yes
		Island-closure interaction (β_3)	0.146 (0.048)	0.052–0.240	No
		Anchovy biomass (β_4)	0.001 (0.001)	0.000–0.002	Yes
		Sardine biomass (β_5)	0.000 (0.001)	−0.002–0.003	Yes
Chick Survival†	Full model (Eqn 2)	Intercept (a)	−5.414 (0.106)	−5.623–−5.210	No
		Closure main effect (β_1)	−0.581 (0.146)	−0.866–−0.296	No
		Island main effect (β_2)	−0.125 (0.126)	−0.371–0.124	Yes
		Island-closure interaction (β_3)	0.344 (0.238)	−0.123–0.811	Yes
		Anchovy biomass (β_4)	−0.074 (0.173)	−0.414–0.265	Yes
		Sardine biomass (β_5)	−0.829 (0.392)	−1.603–−0.067	No
	Final model (β_3 dropped)	Intercept (a)	−5.499 (0.082)	−5.662–−5.338	No
		Closure main effect (β_1)	−0.402 (0.075)	−0.548–−0.256	No
		Island main effect (β_2)	0.023 (0.073)	−0.119–0.165	Yes
		Anchovy biomass (β_4)	−0.201 (0.145)	−0.486–0.082	Yes
		Sardine biomass (β_5)	−1.001 (0.360)	−1.722–−0.308	No
		Eastern Cape (St. Croix and Bird islands)			
Chick Condition	Full model (Eqn 1)	Intercept (a)	0.224 (0.030)	0.164–0.283	No
		Closure main effect (β_1)	0.084 (0.041)	0.004–0164	No
		Island main effect (β_2)	0.138 (0.034)	0.072–0.204	No
		Island-closure interaction (β_3)	−0.168 (0.072)	−0.309–−0.027	No
		Anchovy biomass (β_4)	−0.100 (0.057)	−0.212–0.013	Yes
		Sardine biomass (β_5)	−0.488 (0.186)	−0.851–−0.119	No

†Parameter estimates and CI are shown in (exponential) error space.

Detailed results from the simulation of new condition data

In the original model fit, the 95% credible intervals for the effect sizes of the linear relationships between chick condition and both sardine and anchovy biomass overlapped zero (figure S1). To simplify the simulation of new data, we therefore dropped both biomass terms from the model prior to refitting it to generate the subsample of 1 000 iterations of each MCMC chain to generate used to simulate new chick condition estimates. For the observed data with the biomass estimates excluded, the means (95% credible intervals, CI) were 0.279 (0.239–0.320) for 'Open' years, 0.259 (0.215–0.302) for 'Closed' years at Dassen Island, 0.266 (0.225–0.306) for 'Open' years at Robben Island and 0.378 (0.333–0.424) for 'Closed' years at Robben Island. The percentage effect sizes were −7% at Dassen Island and +42% at Robben Island. These estimates did not differ substantially from the estimates that had sardine and anchovy biomass in the model (compare OB and ONB, figure 3, main text). Note, also, that since we treat year as random effect, we make no assumption about any underlying temporal trend in the data.

The distributions, means and 95% confidence intervals for the simulated data very closely matched the observed data for both islands (figure S2), and the annual means and approximate 95% confidence intervals were appropriate with respect to the observed values (figure 3 main text and figure S3). Furthermore, the estimates for the standard deviations of the nested (month within year)

random effect were 0.395 (95% CI: 0.389–0.401) for the observed dataset, 0.405 (95% CI: 0.401–0.410) with 4 years of simulated data, 0.404 (95% CI: 0.400–0.409) with 7 years and 0.410 (95% CI: 0.401–0.408) with 10 years of simulated data. Plots of the posterior predictive p-values of chick condition by year indicated reasonable model fit (figure S4); we thus were confident that the simulated data sufficiently reflected the effect sizes and variance in the observed dataset. In all cases, the mean closure effect sizes estimated in JAGS remained within the 95% quantiles from 1 000 'nlme' fits, indicating that the JAGS results were robust with respect to sampling variation (figure S6).

With 4 years of simulated data (figure 3, main text), the means (95%CI) were 0.286 (0.258–0.313) for 'Open' years, 0.268 (0.234–0.302) during 'Closed' years at Dassen Island, 0.279 (0.247–0.310) for 'Open' years at Robben Island and 0.402 (0.373–0.432) for 'Closed' years at Robben Island. The percentage effect sizes were −6% at Dassen Island and +44% at Robben Island.

With 7 years of simulated data (figure S3, top panel), the means (95%CI) were 0.279 (0.254–0.304) for 'Open' years, 0.283 (0.257–0.308) during 'Closed' years at Dassen Island, 0.289 (0.264–0.313) for 'Open' years at Robben Island and 0.407 (0.380–0.434) for 'Closed' years at Robben Island. The percentage effect sizes were +1.6% at Dassen Island and +41% at Robben Island.

With 10 years of simulated data (figure S3, bottom panel), the means (95%CI) were 0.283 (0.264–0.303) for 'Open' years, 0.279 (0.256–0.302) during 'Closed' years at Dassen Island, 0.288 (0.267–0.309) for 'Open' years at Robben Island and 0.418 (0.397–0.438) for 'Closed' years at Robben Island. The percentage effect sizes were −1.6% at Dassen Island and +45% at Robben Island

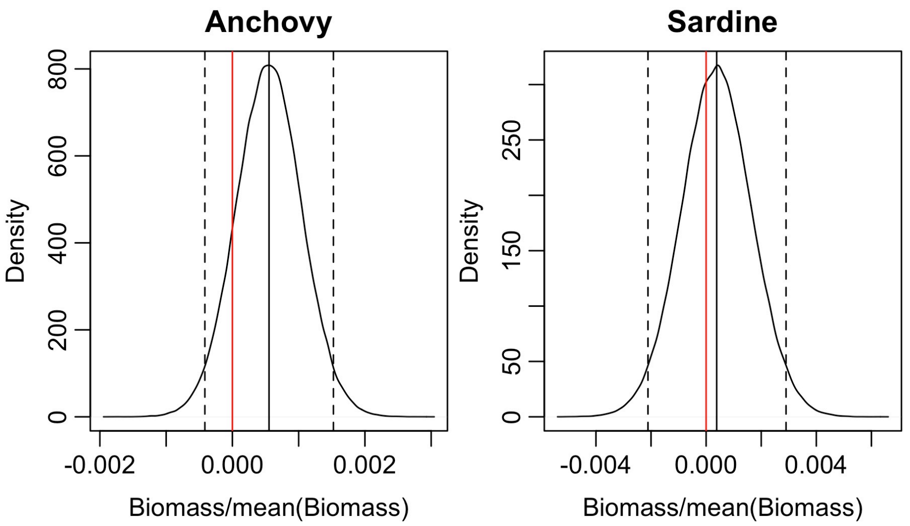

Figure S1. The posterior distributions for the estimated effect of anchvoy biomass (left) and sardine biomass (right) on chick condition at Dassen Island and Robben Island from the maximal model (eqn. 1) with the mean (solid black line) and 95% credible intervals (dashed lines) shown. Values above zero (red line) indicate a positive effect while those below indicate a negative effect.

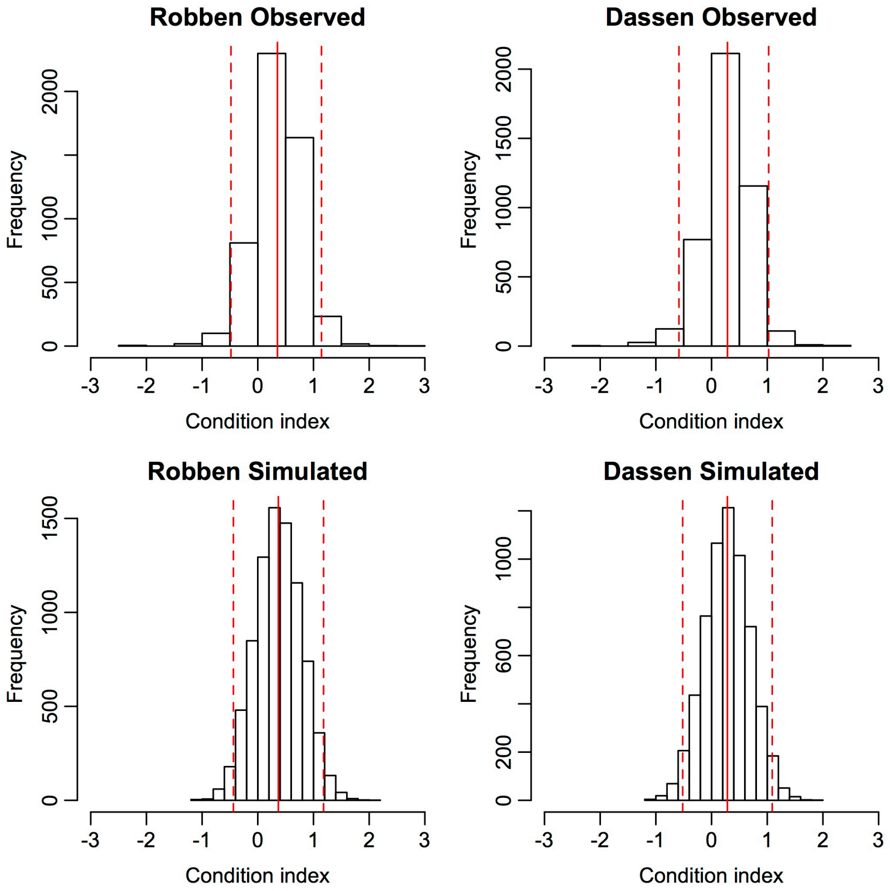

Figure S2. Histograms of the observed 9 436 chick condition measurements (top panels) made at Robben Island (left, 2008–2015) and Dassen Islands (right, 2008–2015) and the 14 161 simulated chick condition measures for the 10-year simulation. Red lines indicate the mean (solid line) and 95% credible intervals (dashed lines) for each distribution; together they show that the simulated distributions correspond to those of the observed data.

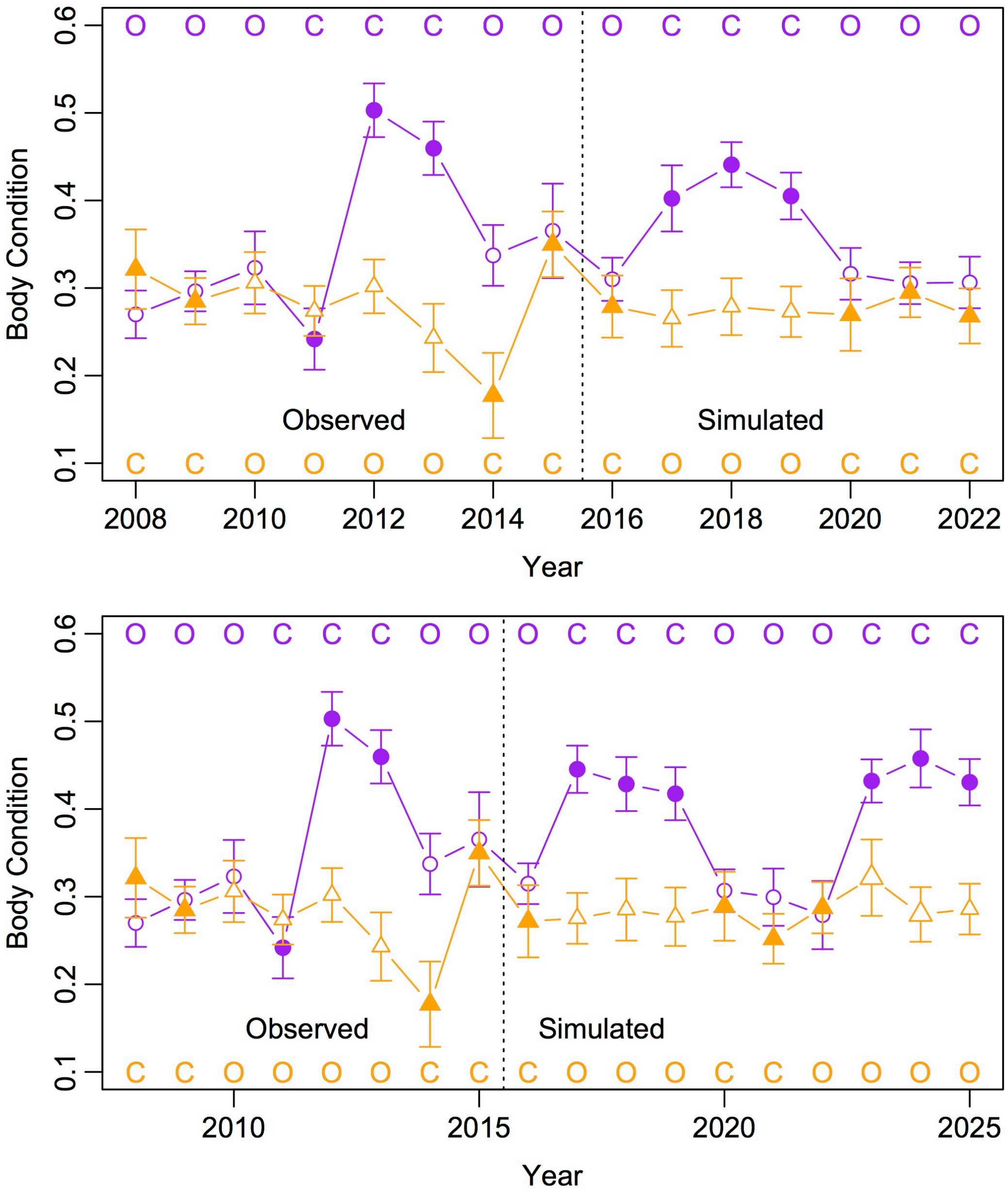

Figure S3. Observed annual means and approximate 95% confidence intervals for chick condition at Robben Island (purple circles) and Dassen Island (orange triangles) for 2008 to 2015 (both panels) and simulated data for seven additional years (2016 to 2022; top panel) and 10 additional years (2016 to 2025; bottom panel). The ‘Closure’ status for each year is indicated by open or closed symbols and at the top (Robben, purple) and bottom (Dassen, orange) of each panel with ‘O’ indicating that fishing was permitted around that island and ‘C’ indicating that fishing was excluded from a 20 km radius around that island. Simulations assumed that the current 3 year closure cycle shown in Table 1 (main text) would continue into the future.

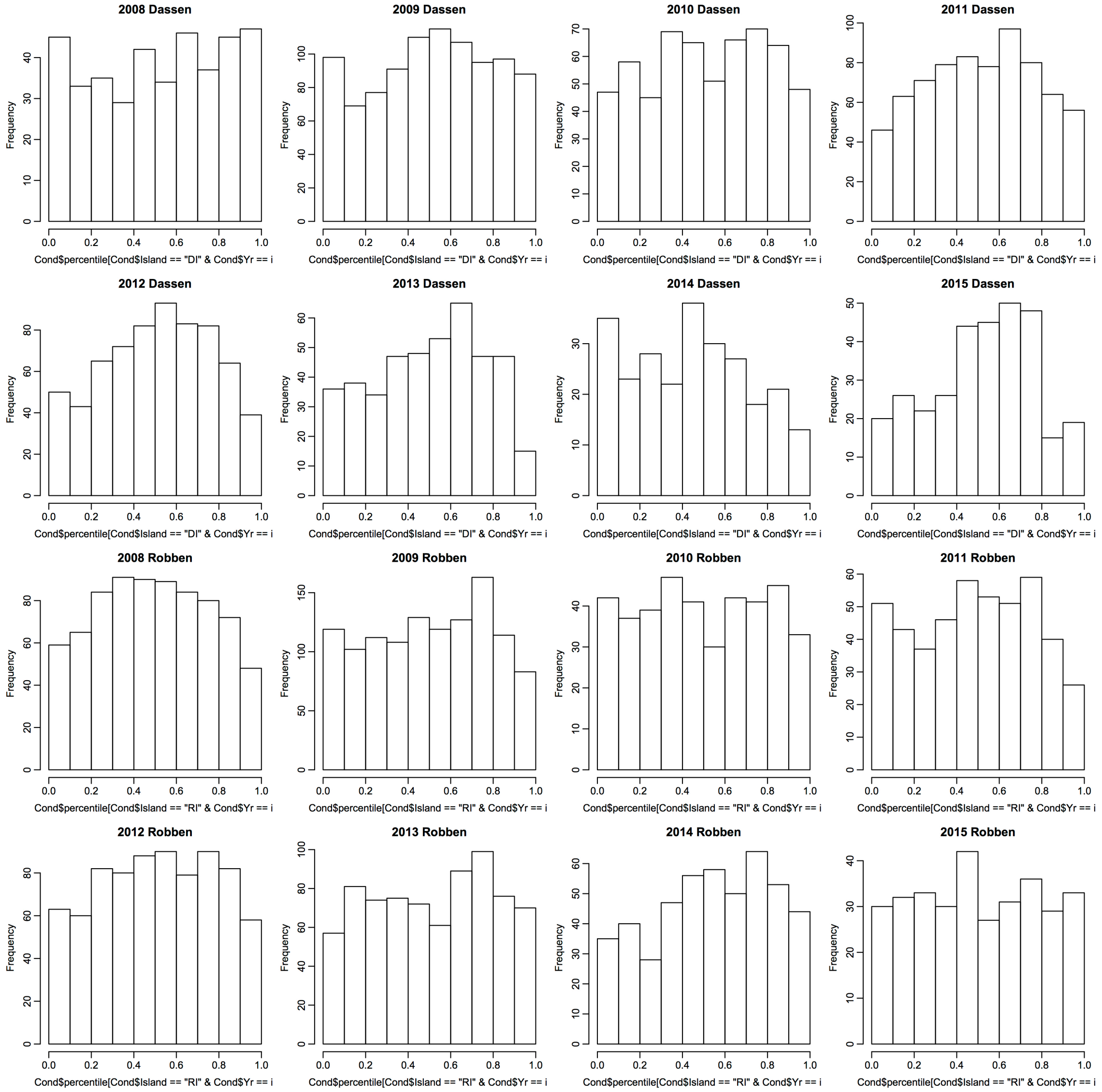

Figure S4. Posterior predictive p-values from the maximal model (eqn. 1, main text) for penguin chick condition by year at Robben Island and Dassen Island, 2008–2015.

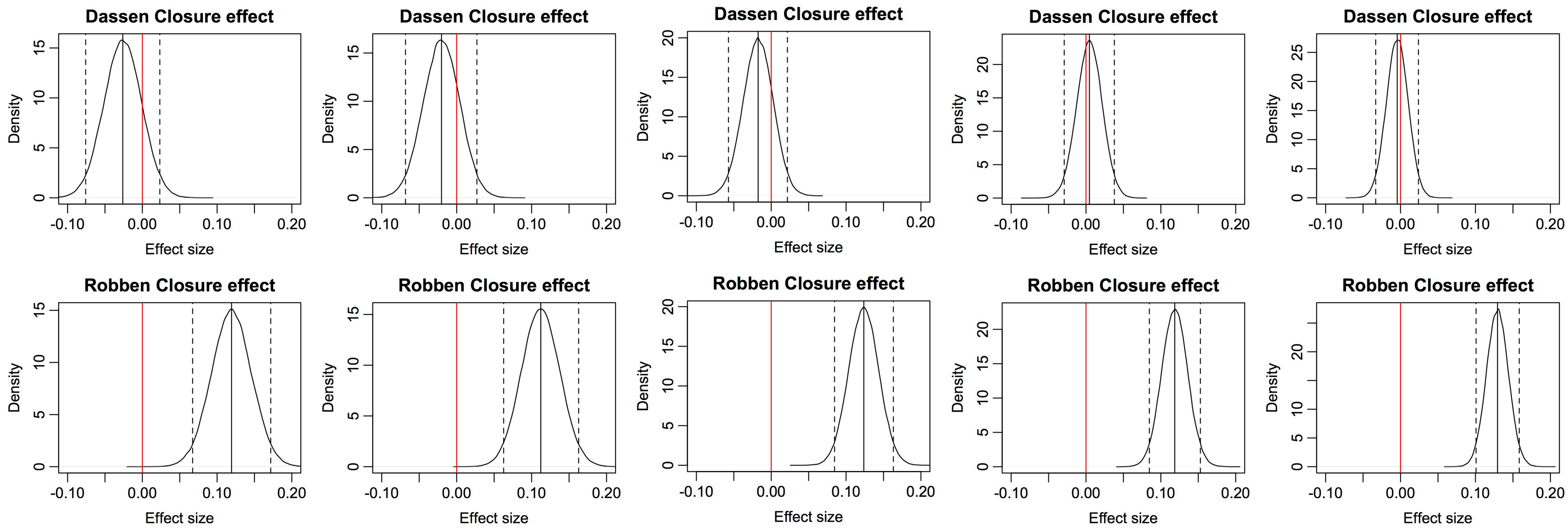

Figure S5. The posterior distributions for the estimated effect of closure to fishing on penguin chick condition at Dassen Island (top row) and Robben Island (bottom row), with the mean (solid black line) and 95% credible intervals (dashed lines). A positive effect size means higher chick condition on average when fishing was restricted from a 20 km radius around each island, a negative effect size the opposite and the zero effect line is shown in red in each panel. The left-hand panel shows the effect sizes for the model fit to the observed data (2008–2015) including sardine and anchovy biomass estimates to account for prevailing environmental conditions; the middle-left panel show the model refitted to the observed data without sardine and anchovy biomass; the center panel shows the model refitted to the observed data plus 4 years of simulated data (see figure 3, main text); the middle-right panel for 7 years of simulated data (see figure S3, top panel) and the right-hand panel for 10 years of simulated data (figure S3, bottom panel).

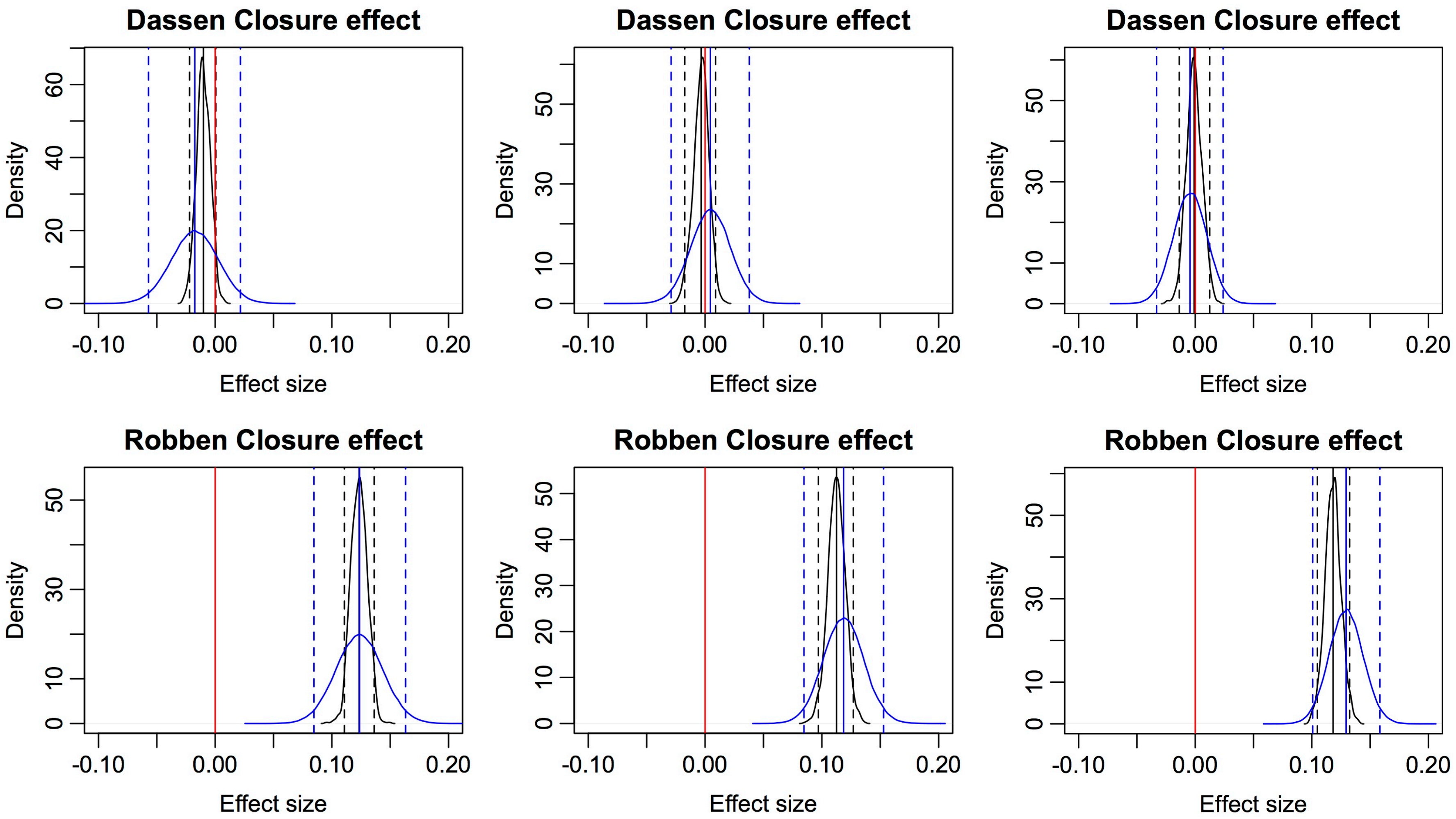

Figure S6: Effect sizes for Dassen Island (top) and Robben Island (bottom) from the JAGS model output using the 4 year (left), 7 year (middle) and 10 year (right) simulated dataset, with the posterior distribution, mean and 95% credible intervals shown (dashed lines) in blue and the mean effect size and 95% quantiles (dashed lines) from 1 000 ‘nlme’ fits to 1 000 randomly generated simulation datasets in black.

Population projections – additional methods and results

Base demographic model: We used a Bayesian projection model with demographic stochasticity and parameter uncertainty to model the changes in the breeding population size at Robben and Dassen Islands between 2004 and 2015 (figure S7). The model approximates the Leslie matrix model with 5 age classes; one juvenile class, three immature adult age classes and one breeding adult class used in previous studies of African penguin demography [3,9]. We assumed a post-breeding census and that all individuals mature at age 4. For the fecundity (*F*) component of the model, $F = P \times f \times R \times \phi_a$, where *P* = breeding probability (assumed to be 1), *f* = the proportion of females in the breeding population (taken to be 0.5) and $R = E \times B \times \phi_e \times \phi_c$, where E = the mean clutch size (1.86 eggs), B = the breeding frequency (1.27 clutches per annum), ϕ_e = egg survival (0.548) and ϕ_c = chick survival (see table S2). Adult survival (ϕ_a) was deterministic, and set at 0.743 [3].

For the base run, ϕ_c was assumed to be sampled with uncertainty around the mean (± SD) value estimated for all 'Open' years at both islands (see table S2). Juvenile survival (ϕ_j) was also assumed to be sampled with uncertainty around a mean (± SD) value of 0.194 (± 0.117) based on the well-estimated (SE ≤ 1.1) annual values for Robben and Dassen Island between 2001/02 and 2011/12 [9,11]. Both of these means and SDs were mapped to beta distributions for inclusion in the model.

We used a starting population based on the combined Robben and Dassen island populations in 2004 (33,425 breeding pairs [12]) in the adult age class (see table S2 for starting age distribution) and ran the models using three MCMC chains of 225 000 samples (burn-in of 25 000, no thinning). We used beta prior distributions for all stochastic survival rates and binomial and Poisson distributions to map the number of individuals in each of the five states from year *t* to *t*+1 (thus allowing for demographic stochasticity). The model was run for *t* = 27 years (thus, simulating the population trajectory from 2004 to 2030) and the population growth rate (λ) was calculated using *t* = 6 to *t* = 27 to ensure convergence at the stable-age distribution. This mean λ value (± 95% CI) was then used to predict a modelled population for 2004 to 2015 (± 95% CIs) and compared to the observed population trajectory.

Linking chick body condition to juvenile survival (ϕ_j): In the absence of species-specific data to link changes in body condition to either chick (ϕ_c) or juvenile survival (ϕ_j), we first mapped the percentage change in condition directly as a percentage change in ϕ_j by assuming linear proportionality. Studies on other penguin species suggest this to be a reasonable first assumption in most years [13,14]. We then modelled improvements in chick body condition as improvements in ϕ_j through two steps to link (1) observed relationships between mass at fledging and first-year survival in Macaroni penguins [13,14] to (2) an observed relationship between mass at fledging and body condition in 39 African penguin fledglings measured in 2012 and 2013 [9] (figure S8). To do this we first calculated standardised fledging mass (m') for both species as:

$$m'_i = m_i - \left(\frac{\overline{m}}{s_m}\right)$$

(eqn. S1)

where m_i is each mass measurement, $\bar{m}$ the mean mass and s_m the standard deviation in the dataset for each species.

Then for the first step (1), we used the modelled relationships between m' and survival (s) for each of seven years (2003–2009) from Figure 4 in [14] to calculate a mean survival rate as a function of m' (figure S8A). As there were seven survival rates estimated for each measure of m', we treated these as repeated measures and implemented a linear-mixed model structure, with the vector of standardised mass measurements as the predictors, the survival rate estimates (on the logit scale) as the responses and random intercepts modelled for each mass measurement. This model took the form:

$$logit(s_{i,j}) = \alpha_1 + b_j + \beta_1 m'_j + \varepsilon_{i,j}$$
$$i = 1, \dots, n_{i,j} = 707,\ \ j = 1, \dots, 101,\ \ b_j \sim N(0, \sigma^2),\ \ \varepsilon_{i,j} \sim N(0, \sigma^2)$$

(eqn. S2)

where α is the intercept; β the estimated slope coefficient for the linear effect of standardised mass on logit(survival); $s_{i,j}$ the survival rates associated with each mass measurement j; m'_j the covariate vector of standardised mass measurements; b_j the random effect for the repeated survival measures; $\varepsilon_{i,j}$ the residual error; and the σ's are estimated from the data.

For the second step (2), we fit a linear model of the form:

$$m'_i = \alpha_2 + \beta_2 c_i + \varepsilon_i$$
$$i = 1, \dots, n_i = 39,\ \ \varepsilon_i \sim N(0, \sigma^2)$$

(eqn. S3)

where α is the intercept; β the estimated slope coefficient for the linear effect of body condition (c) on standardised mass (m') and ε_i the residual error with σ estimated from the data (figure S8B). We fit both models using MCMC estimation in JAGS as part of the population projection models outlined in the main text. We used uninformative priors, with $N(0, 10^{-7})$ for means and $U(0{,}100)$ for standard errors (σ) as outlined in the main text.

We then incorporated these estimates and their associated uncertainties into the likelihoods for the population projection models by first predicting standardised mass values in 'Open' ($\widehat{m}_o$) and 'Closed' ($\widehat{m}_c$) based on the mean chick condition from eqn 1 in the main text in 'Open' ($\bar{y}_o$) and 'Closed' ($\bar{y}_c$) years at Robben Island, then used these $\widehat{m}$ values to predict juvenile survival in 'Closed' years $\hat{s}_{j,c}$. Because we wanted to model the impact of improved chick condition above the observed ϕ_j = 0.194, we then corrected these survival rates so that the observed chick condition in 'Open' years at Robben Island corresponded to a modelled $\hat{s}_{j,c}$ = 0.194 (figure S8C):

$$\widehat{m}_o = \alpha_2 + (\beta_2 \times \bar{y}_o)$$

and

$$\widehat{m}_c = \alpha_2 + (\beta_2 \times \bar{y}_c)$$

then

$$\hat{s}_{j,c} = (\exp(\alpha_1 + (\beta_1 \times \widehat{m}_c))/(1 + \exp(\alpha_1 + (\beta_1 \times \widehat{m}_c))))$$
$$-((\exp(\alpha_1 + (\beta_1 \times \widehat{m}_o))/(1 + \exp(\alpha_1 + (\beta_1 \times \widehat{m}_o)))) - \emptyset_j)$$

(eqn. S4)

Because these regression relationships were estimated in JAGS and incorporated directly into the population projection models, the uncertainty in the underlying relationships (1 and 2 above) could be carried over and explicitly taken into account. The linear proportionality assumption both overestimated the impact of condition on ϕ_j and fell within the 95% credible intervals for our predicted relationship (figure S8C); we therefore used our predicted relationship for all subsequent models.

Table S2. Demographic parameters used in the base population projection model (see figure 2) and their sources.

Parameter	Parameter value	Source
Adult survival[1] (ϕ_a)	0.743	[3,11]
Juvenile survival (ϕ_j)	0.194 (SD = 0.117)	[11]
Mean clutch size (*E*)	1.86	[15,16]
Egg survival (ϕ_e)	0.55	[3,15]
Chick survival (ϕ_c)	0.736 (SD = 0.017)	This paper, eqn. 2
Clutches per annum (*C*)	1.27	[15,16]
Fecundity (*F*)[2]	($E \times \phi_e \times \phi_c \times C$)	-
Assumed sex ratio	1:1	[3]
Starting populations[3]:		
Juveniles	2060	-
Immature 1	2000	-
Immature 2	2000	-
Immature 3	2000	-
Adults (breeders)	33,425	[12]

Notes: 1. Annual survival in the three immature and the one breeding adult states were equal as no difference has been detected in a recent multistate capture-mark-recapture (CMR) analysis for this species [11]. 2 The model/scenario specific fecundity (*F*) = $E \times \phi_e \times \phi_c \times C$ for each model, following [3]. 3. The starting population for the adult state is the number of breeding pairs counted in the 2004 annual census; the starting populations for the other states were approximated based on the stable stage distribution at convergence.

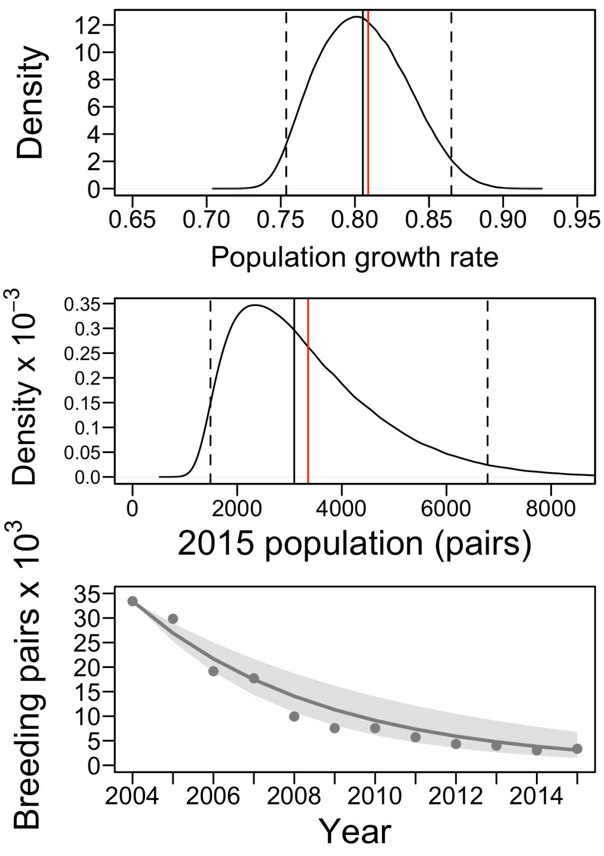

Figure S7. Top: The distribution of the population growth rate estimates (λ) for the base demographic model (see Materials and Methods, in the main text), with the mean (solid black line) and 95% Bayesian credible intervals (CI; dashed lines). The deterministic λ value from an equivalent Leslie matrix model (λ = 0.809) is shown as a solid red line. Middle: The posterior distribution for the 2015 modelled population at Robben and Dassen islands in pairs with the mean (solid black line) and 95% BCIs (dashed lines) and observed 2015 popualtion (solid red line) shown. Bottom: The observed (grey circles) and modelled mean (grey lines ± 95% CI shown as a grey polygon) population projection for Robben and Dassen islands from 2004 to 2015.

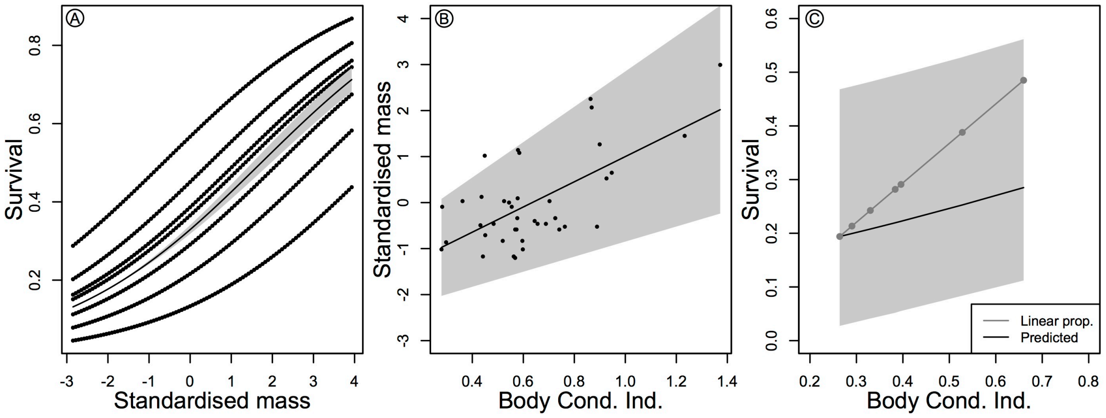

Figure S8. (A) The relationship between first-year survival and standardised mass in Macaroni penguins over seven seasons (thick dotted lines) from [13,14] and the modelled mean relationship (thin black line) and 95% credible intervals (grey polygon) based on equation S2; (B) The relationship between chick body condition at fledging and standardised mass in 39 African penguins from [9] and the modelled mean relationship (solid black line) and 95% credible intervals (grey polygon) based on equation S3; (C) the resulting relationship (black line), with uncertainty (95% credible intervals, grey polygon), used to predict the relationship between improvements in chick condition and juvenile survival (ϕ_j) in the population projection models. The comparison with linear proportionality (grey line and points), which falls within the 95% CIs for the predicted relationship, is shown.

Literature cited:

1. Sherley RB, Waller LJ, Strauss V, Geldenhuys D, Underhill LG, Parsons NJ. 2014 Hand-rearing, release and survival of African penguin chicks abandoned before independence by moulting parents. *PLoS ONE* **9**, e110794. (doi:10.1371/journal.pone.0110794)

2. Sherley RB, Underhill LG, Barham BJ, Barham PJ, Coetzee JC, Crawford RJM, Dyer BM, Leshoro TM, Upfold L. 2013 Influence of local and regional prey availability on breeding performance of African penguins *Spheniscus demersus*. *Mar. Ecol. Prog. Ser.* **473**, 291–301. (doi:10.3354/meps10070)

3. Sherley RB, Winker H, Altwegg R, van der Lingen CD, Votier SC, Crawford RJM. 2015 Bottom-up effects of a no-take zone on endangered penguin demographics. *Biol. Lett.* **11**, 20150237. (doi:10.1098/rsbl.2015.0237)

4. Seddon PJ, van Heezik YM. 1993 Behaviour of the jackass penguin chick. *Ostrich* **64**, 8–12. (doi:10.1080/00306525.1993.9634188)

5. Sherley RB, Barham BJ, Barham PJ, Leshoro TM, Underhill LG. 2012 Artificial nests enhance the breeding productivity of African Penguins (*Spheniscus demersus*) on Robben Island, South Africa. *Emu* **97**, 97–106. (doi:10.1071/MU11055)

6. Kruschke J. 2015 *Doing Bayesian data analysis: A tutorial with R, JAGS, and Stan. 2nd edn.* New York, NY: Academic Press.

7. Hutchings L, Jarre A, Lamont T, van den Berg M, Kirkman SP. 2012 St Helena Bay (southern Benguela) then and now: muted climate signals, large human impact. *Afr. J. Mar. Sci.* **34**, 559–583. (doi:10.2989/1814232X.2012.689672)

8. Crawford RJM *et al.* 2011 Collapse of South Africa's penguins in the early 21st century. *Afr. J. Mar. Sci.* **33**, 139–156. (doi:10.2989/1814232X.2011.572377)

9. Sherley RB, Ludynia K, Dyer BM, Lamont T, Makhado AB, Roux J-P, Scales KL, Underhill LG, Votier SC. 2017 Metapopulation Tracking Juvenile Penguins Reveals an Ecosystem-wide Ecological Trap. *Curr. Biol.* **27**, 563–568. (doi:10.1016/j.cub.2016.12.054)

10. Weller F, Sherley RB, Waller LJ, Ludynia K, Geldenhuys D, Shannon LJ, Jarre A. 2016 System dynamics modelling of the Endangered African penguin populations on Dyer and Robben islands, South Africa. *Ecol. Model.* **327**, 44–56. (doi:10.1016/j.ecolmodel.2016.01.011)

11. Sherley RB, Abadi F, Ludynia K, Barham BJ, Clark AE, Altwegg R. 2014 Age-specific survival and movement among major African Penguin *Spheniscus demersus* colonies. *Ibis* **156**, 716–728. (doi:10.1111/ibi.12189)

12. Sherley RB, Barham PJ, Barham BJ, Crawford RJM, Dyer BM, Leshoro TM, Makhado AB, Upfold L, Underhill LG. 2014 Growth and decline of a penguin colony and the influence on nesting density and reproductive success. *Popul. Ecol.* **56**, 119–128. (doi:10.1007/s10144-013-0394-1)

13. McClung MR, Seddon PJ, Massaro M, Setiawan AN. 2004 Nature-based tourism impacts on yellow-eyed penguins Megadyptes antipodes: does unregulated visitor access affect fledging weight and juvenile survival? *Biol. Conserv.* **119**, 279–285. (doi:10.1016/j.biocon.2003.11.012)

14. Horswill C, Matthiopoulos J, Green JA, Meredith MP, Forcada J, Peat H, Preston M, Trathan PN, Ratcliffe N. 2014 Survival in macaroni penguins and the relative importance of different drivers: individual traits, predation pressure and environmental variability. *J. Anim. Ecol.* **83**, 1057–1067. (doi:10.1111/1365-2656.12229)

15. Shannon LJ, Crawford RJM. 1999 Management of the African penguin *Spheniscus demersus* – insights from modelling. *Mar Ornithol* **27**, 119–128.

16. Crawford RJM, Shannon LJ, Whittington PA. 1999 Population dynamics of the African penguin *Spheniscus demersus* at Robben Island, South Africa. *Mar. Ornithol.* **27**, 139–147.

Electronic Supplementary Material S2

JAGS code used to fit the model to estimate the effect of island closure on chick condition (eqn. 1):

```
model {
    # The data are condition indices for each chick ('cond')
    # measured within a month ('month') and year ('year') which are specified
    # as hierarchal random effects.
    # 'closure' indicates whether the condition measure is from open years
    # (closure=0) or closed years (closure=1)
    # biomass estimates of prey (anchovy, 'anch', and sardine 'sard') are
    # added as controls for environmental variability

##############################
# Priors and constraints
##############################

 mu.oD~dnorm(0, 0.0000001) # Mean for hyperparameter for nested random effect
 sigma.int~dunif(0, 100) # SD for hyperparameter for nested random effect
 tau.int <- pow(sigma.int, -2)

    for (j in 1:J) {
    for (k in 1:K) {
    alpha[j,k]~dnorm(mu.oD, tau.int) # Random intercepts
    }#j
    }#k

beta.close ~ dnorm(0, 0.0000001)          # diff. between Open and Closed
beta.anch ~ dnorm(0, 0.0000001)        # effect of anchovy biomass
beta.sard ~ dnorm(0, 0.0000001)        # effect of sardine biomass
beta.island ~ dnorm(0, 0.0000001)        # effect of island
beta.inter ~ dnorm(0, 0.0000001)        # island-closure interaction
sigma ~ dunif(0, 100)
tau.resid <- pow(sigma, -2)   # Residual Standard Error

##############################
# Likelihood
##############################

for (i in 1:n)  # for each of n chicks measured
    {
    cond[i] ~ dnorm(mu[i], tau.resid)
    mu[i] <- alpha[year[i],month[i]] + beta.close*closure[i] +
             beta.island*island[i] + beta.anch*anch[i] + beta.sard*sard[i] +
             beta.inter*island[i]*closure[i] # linear predictor

    cond.new[i] ~ dnorm(mu[i], tau.resid)
    #res[i] <- cond[i]-mu[i]
    #res.new[i] <- cond.new[i]-mu[i]
    }

##############################
# Derived parameters
##############################

mu.cD <- mu.oD + beta.close       # mean condition for Closed at Dassen
mu.oR <- mu.oD + beta.island      # mean condition for Open at Robben
mu.cR <- mu.oD + beta.close + beta.island + beta.inter  # mean condition for
                                                       # Closed to fishing at Robben
bC.bI <- beta.close + beta.inter # Effect size for Closure at Robben
} # end model
```

JAGS code used to fit the model to estimate the effect of island closure on chick survival (eqn. 2):

```
model{

###############################
# Likelihood
###############################

for (i in 1:N) {
  isCensored[i] ~ dinterval(t[i],t.cen[i])
  t[i] ~ dexp(mu[i])

  mu[i] <- exp(alpha + beta.island*island[i] + beta.close*close[i] +
  beta.inter*island[i]*close[i] + beta.sard*sard[i] + beta.anch*anch[i] +
  b[year[i],nest[i]])
  } # i

###############################
# Priors and constraints
###############################
  alpha ~ dnorm(0.0, 0.0001)
  beta.island ~ dnorm(0.0, 0.0001)
  beta.close ~ dnorm(0.0, 0.0001)
  beta.inter ~ dnorm(0, 0.0001)        # effect of island-closure interaction
  beta.sard ~ dnorm(0.0, 0.0001)
  beta.anch ~ dnorm(0.0, 0.0001)

  for (y in 1:Y) {
  for (c in 1:C) {
  b[y,c] ~ dnorm(0.0, tau) # Nested frailty (random effect)
  } #y
  } #c

  sigma ~ dunif(0, 100)
  tau <- pow(sigma, -2) # s.d. of random effects


###############################
# Derived parameters
###############################

mu.oD <- 1/exp(alpha)   # Mean time to death (MTD), Dassen in Open years
mu.cD <- 1/exp(alpha + beta.close)# MTD, Dassen in Closed years
mu.oR <- 1/exp(alpha + beta.island) # MTD, Robben in Open years
mu.cR <- 1/exp(alpha + beta.close + beta.island + beta.inter) # MTD, Robben
                                                               # in Closed years
bC.bI <- beta.close + beta.inter   # Diff. for Island/Closure at Robben

  S.DO <- exp(-exp(alpha))^74 # Survival rate @74 days for Dassen, Open years
  S.DC <- exp(-exp(alpha + beta.close))^74 # Survival, Dassen, Closed years
  S.RO <- exp(-exp(alpha + beta.island))^74 # Survival, Robben, Open years
  S.RC <- exp(-exp(alpha + beta.close + beta.island + beta.inter))^74
        # Survival, Robben, Closed years

  } # end model
```
